# Postoperative Telephone-Based Questionnaire on Quality of Life after Robotic-Assisted Laparoscopic Hysterectomy versus Conventional Total Laparoscopic Hysterectomy

**DOI:** 10.3390/jcm9092849

**Published:** 2020-09-02

**Authors:** Mohamed Elessawy, Sarah Schneekloth, Veronika Günther, Nicolai Maass, Liselotte Mettler, Ibrahim Alkatout

**Affiliations:** 1 Department of Gynecology and Obstetrics, University Hospitals Schleswig-Holstein, Campus Kiel, 24105 Kiel, Germany; Veronika.Guenther@uksh.de (V.G.); Nicolai.maass@uksh.de (N.M.); profmettler@gmx.de (L.M.); Ibrahim.Alkatout@uksh.de (I.A.); 2 Department of Gynecology and Obstetrics, University Hospitals Schleswig-Holstein, Campus Luebeck, 23562 Luebeck, Germany; Sarah.Schneekloth@uksh.de

**Keywords:** robotic surgery, sexuality, laparoscopic hysterectomy, learning curve, quality of life, counseling, patient-doctor-relationship

## Abstract

*Aim*: The objective of the study was to evaluate the benefits of robotic-surgery for hysterectomy compared to conventional laparoscopy for benign indications. A specially prepared telephone-based questionnaire was used postoperatively. *Method*: All women (*n* = 155) undergoing total laparoscopic hysterectomy for benign indications either by the robotic-assisted procedure (RALH) or conventional laparoscopy (CL) between 1 January 2013 and 31 December 2017 at the Department of the Gynecology, University Hospitals, Campus Kiel, Germany, were eligible for analysis. Intra-operative and postoperative parameters affecting the patients’ quality of life were assessed by a telephone-based questionnaire. The latter addressed postoperative pain, limitations of basic hygiene, daily activity, active pursuit of hobbies, sexual intercourse, and days of sick leave. All patients received the questionnaire by post at least three weeks prior to being contacted on the phone. *Results*: 78% of the contacted patients responded to the questionnaire; 96% (*n* = 115) of the patients said they would recommend the operation to other patients. Both groups needed 42 days to resume their regular hobbies. In whole 90.8% (*n* = 108) were total satisfied with the cosmetic result of the abdominal incision; the numbers in the respective groups were 80% (80% *n* = 36) in RALH and 97.3% (*n* = 72) in CL. The difference was significant on the Chi-square test (*p* = 0.002). 5% (*n* = 7) were dissatisfied with the scar (13.3%; *n* = 6) in the RALH group, and 1.4% (*n* = 1) in CL. In all 1.7 % of patients were dissatisfied with the position of the incisions; the respective numbers were 4.4 % (*n* = 2) in the RALH group and no patient in the CL group. 33% of patients experienced no limitations in regard of sexual intercourse after the operation. The median number of days taken to resume sexual intercourse after the operation was 56 days in the CL group, and 49 days in the RALH group. Nearly 30% (*n* = 25) were hesitant to resume intercourse. The median operating time was 145 min in the RALH group, which was significantly longer than the 117 min taken in the CL group (*p* < 0.001). *Conclusions*: The RALH procedure was associated with some minor advantages for the patients according to the results, however it does not have major significant advantages, especially in regard of early restoration of sexual function, while the CL shows shorter operating times and similar limitation. Postoperative counseling of patients should be aligned to their fears and expectations in regard of sexual function.

## 1. Introduction

Hysterectomy is one of the most commonly performed surgical procedures in women. At all university hospitals in Germany, about 4338 hysterectomies were performed for benign indications in 2016.

Although hysterectomy is a standard treatment for gynecologic malignancies, many hysterectomies are performed for benign gynecologic disease [1]. In the last decade, a number of national trends have influenced surgical practices [2,3]. The rapid developments of the technology in the instruments and telemedicine have influenced the development of surgery. Vaginal hysterectomy has been performed for several decades. If feasible, it is still recommended as the treatment of choice by the German national guideline and the most recent Cochrane analysis [4]. Laparoscopically-assisted hysterectomy and conventional total laparoscopic hysterectomy (CL) have been used since the 1990s [5]. Minimally invasive surgical techniques are now used for many procedures. More recently, robotic assisted laparoscopic hysterectomy (RALH) has also become an established technique [6].

The potential benefits of robotic-assisted laparoscopic surgery include a larger range of motion with the instruments, three-dimensional stereoscopic visualization, and improved ergonomics during surgery [7]. Unlike procedures such as prostatectomy or colorectal surgery, for which robotic assistance is the sole alternative to the open approach, both laparoscopic and vaginal hysterectomies are widely performed without significant problems [8]. However, the benefits of robotic-assisted laparoscopic surgery are still not clearly established. In women with benign disease, RALH did improve outcomes, was associated with longer operating times and higher costs compared to hysterectomy by CL [9].

Prospective trials comparing CL with RALH have failed to demonstrate significant improvements in clinical outcomes in women with benign gynecologic disease [10,11]. Therefore, we conducted a telephone-based postoperative survey on quality of life and convalescence among patients undergoing RALH with a matched cohort with conventional total laparoscopic hysterectomy to assess the benefits of RALH in comparison to CL for benign indications. The purpose was to determine whether the variables addressed in the questionnaire play a significant role in the treatment choice based on a shared decision-making. Furthermore, we determined the patients’ level of satisfaction with abdominal scars after RALH and their limitations in regard of sexual intercourse after surgery.

### Objective 

The objective was to compare peri- and postoperative outcomes, focusing on the patients’ satisfaction with the treatment and their limitations, including: convalescence, sick leave, sexual intercourse, perioperative morbidity, postoperative pain, the number and positions of scars.

## 2. Method

All women undergoing total laparoscopic hysterectomy for benign indications by RALH between 1 January 2013 and 31 December 2017 at the Department of the Gynecology, University Hospitals, Campus Kiel, Germany, were eligible for analysis. Patients who underwent TLH by CL during the same period, matched for age, indication for hysterectomy, comorbidities, body mass index, uterine weight and histopathology, served as controls. The study was approved by the ethics committee of the University of Kiel (574/17). Written informed consent was obtained from all patients.

### 2.1. Design of Questionnaire

Postoperative parameters affecting the patients’ quality of life were addressed in a postoperative telephone interview. The latter was focused on postoperative pain, limitations of basic hygiene, daily activities, active pursuit of hobbies, sexual intercourse, and days of sick leave.

The questionnaire (Appendix A) was designed on the basis of the following: (a) a validated German version of King’s health questionnaire for assessing quality of life in women [12], (b) the EQ-5D (a standardized health-related quality of life questionnaire developed by the EuroQol Group to provide a simple generic measure of health for clinical and economic appraisal) [13], and (c) the validated Female Sexual Function Index (FSFI; a brief multidimensional scale for assessing sexual function in women) [14].

Postoperative pain scores were recorded at one and four weeks after the operation. Pain was determined on a numeric rating scale (NRS) as recommended by the Initiative on Methods, Measurement and Pain Assessment in Clinical Trials (IMMPACT) [3]. Patients rated their pain on a scale from 0 to 10 (0 = no pain; 10 = worst imaginable pain). To avoid bias resulting from different cognitive levels, we used construct-specific questions for a satisfaction scale from 1 to 6 [15]; 1 indicated extreme satisfaction and 6 indicated dissatisfaction.

All patients received the questionnaire by post at least three weeks before they were contacted by phone; an appointment for the telephone interview was suggested in the questionnaire sent by post. We contacted 155 patients and received a reply from 122 (78.7%). Three patients were excluded due to the lack of ability to answer the questioner due to limited cognitive functions. The ethics committee to avoid any violation to the patient’s privacy suggested a three weeks interval between the sending the mail and contacting the patients per telephone and we were only allowed to contact the patients per phone after receiving the written informed consent per Post.

### 2.2. Telephone Interview

Based on former QOL investigations of our study group [16], the telephone interview was designed as an interactive measure. Data published by the University of Heidelberg, Germany, showed that responses to an interactive interview are more explicit and critical, which makes the interview more suitable for quality control [17]. We minimized the influence of disruptive factors by conducting a standardized interview and avoiding any open conversation with the patient. To avoid any bias, the standardized telephone interviews were conducted by the same operator (SS).

### 2.3. Material

The study was designed to investigate parameters affecting the clinical outcome of RALH and CL, list the various indications for the procedures, the development of the procedures, compare complication rates and outcomes, and their association with the route of surgery.

Cases were also matched for the surgeon, who performed 56 operations by RALH and 99 by CL. The surgeon is a highly trained surgeon certified as MIC III surgeon for minimally invasive surgery held by the German society of gynecological laparoscopic surgery (*Arbeitsgemeinschaft Gynäkologische Endoskopie*, AGE). The surgeon who performed the procedures had attended a robotic training course and was then proctored by experienced robotic surgeons.

RALH was performed using the four-arm da Vinci surgical system (Intuitive Surgical, Sunnyvale, CA, USA). Patient data were entered into a Microsoft Excel database. The total operating times were derived from the operating theatre database for TLH by CL, and the da Vinci surgical procedure database for RALH. The total operating time included: (a) installation of the uterine manipulator; (b) ‘skin-to-skin’ operating time, defined as the time from the first skin incision to skin closure and (c) console time, defined as the time from the start of operating the console to de-docking of the surgical cart.

Intra- and postoperative complications until 20 weeks after surgery and the length of hospital stay were noted. The Clavien Dindo classification system for grading surgical complications was used [18]. Complications were further classified into intraoperative (urinary tract, gastrointestinal tract, vascular injury) and postoperative complications (revision, wound healing, thromboembolic events, mortality, systemic inflammatory response associated with fever, urinary tract infection, Clostridia infection, transient paresthesia, and hemoptysis).

### 2.4. Statistical Analysis

Data were entered on a spreadsheet in the computer. The IBM SPSS statistics program (IBM Corp IBM, NY, USA) was used to log and analyze the data. Professional statistical guidance was provided by the Medistat GmbH office. Differences between groups in regard of the analyzed parameters were subjected to statistical analysis. The following tests were used: (a) Chi-square test for the analysis of differences between two proportions, (b) T-test to determine the significance of differences between two proportions or percentages, and (c) The Mann-Whitney U-test and Wilcoxon’s signed rank test to compare one quantitative value between two groups of patients. Demographic and surgical data were analyzed by analysis of variance (ANOVA), Kruskal-Wallis, Chi-square, or Fisher’s test. A p value less than 0.05 was considered statistically significant. We also used the Lilliefors significance correlation when a significant correlation R-value of more than 0.2 was considered to be statistically correlated.

## 3. Results

### 3.1. Patient Characteristics

One-hundred and fifty-five women underwent hysterectomy during the study period. Ninety-nine women had a conventional total laparoscopic hysterectomy (CL) and 56 underwent a robotic-assisted laparoscopic hysterectomy (RALH).

Indications for surgery were the following in the CL and RALH groups: uterine myomas in 43 (43.4%) and 28 (50%) patients, respectively; premalignant lesions such as diffuse hyperplasia of the endometrium and intracervial neoplasia in 26 (26.3%) and nine (16.1%) patients, respectively; endometriosis in 17 (17%) and 13 (23.2%) patients, respectively; abnormal uterine bleeding in nine (9.1%) and six (10.7%) patients, respectively; and other indications such as completed family planning, carcinophobia and transgender transformation in four (4.9%) and one (1.8%) patients, respectively.

The main indications for TLH by CL and RALH were similar; no statistical difference was noted between groups. The same was true of the patients’ age and body mass index. We registered concomitant gynecologic procedures, including adhesiolysis, salpingo-oophorectomy, ureterolysis, endometriosis resection, and others such as filling the bladder with colored dye, suturing the bladder wall, suturing the serosa of the intestine, cyst enucleation, adhesion prophylaxis, transient abdominal ovariopexy, and colposuspension. Demographic data and indications for surgery are summarized in Table 1.

### 3.2. Operating Time

**Figure 1:**
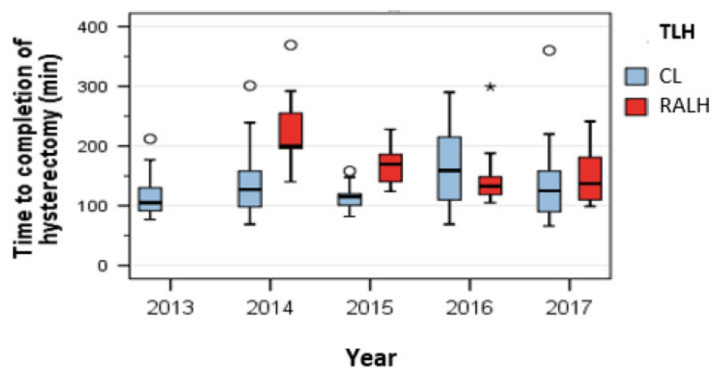
Operating times during the study: the shortest operating time for RALH was noted in 2016.

The median operating time was significantly longer in the RAHL group (145 min) than in the CL group (117 min) (*p* < 0.001). The operating time for RALH fell markedly in 2016: 132.50 min was the shortest operating time registered for RALH during the study. The median time taken to perform CL in 2016 was 159.00 min (Figure 1).

### 3.3. Learning Curve

**Figure 2:**
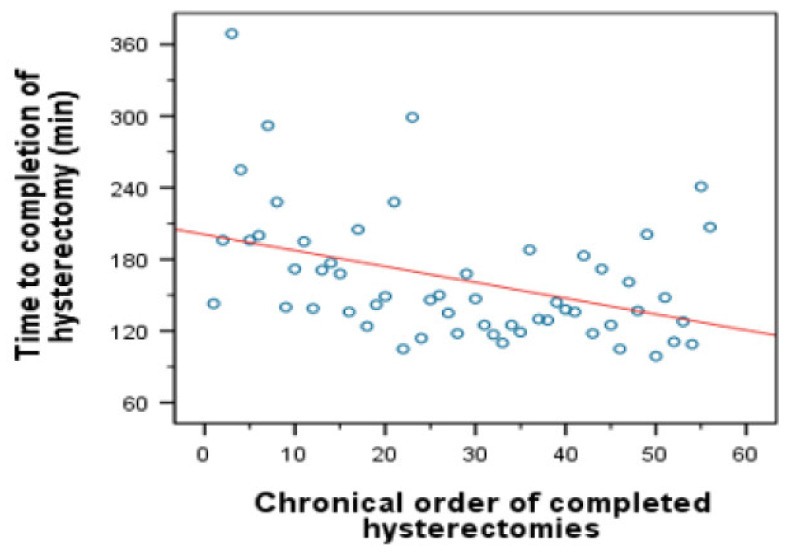
Linear regression for operating time.

In the RALH group we noted a significant fall in median operating time after the first 30 cases (Figure 2). The mean operating time fell by 38.5 min from 168 min (cases 1–30) to 129.50 min (cases 30–56).

**Figure 3:**
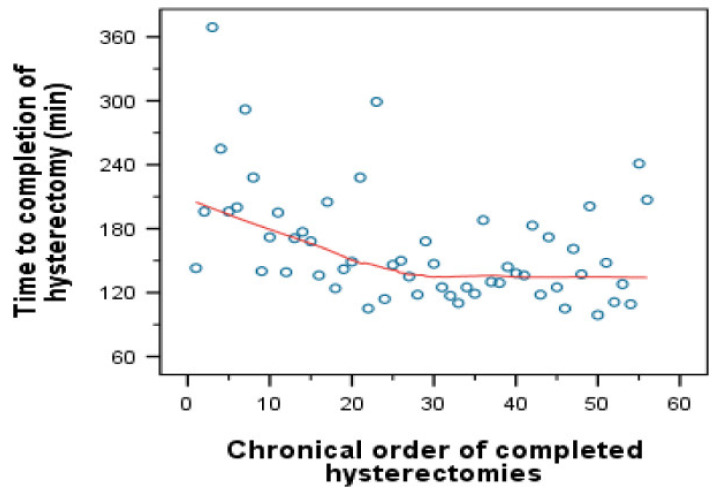
The linear regression for operating time according to Loess.

Linear regression showed a significant reduction until case number 30 (*p* = 0.012), and no regression thereafter (*p* = 0.108) (Figure 3). No notable learning curve was observed for conventional laparoscopy because the surgeon had performed more than 1.000 surgical laparoscopies before the investigation was started.

### 3.4. Intra- and Postoperative Complications

Two patients in the RALH group and four patients in the CL group sustained iatrogenic bladder injuries (*p* > 0.999). Although there was no significant difference between groups, the total number of bladder injuries was rather high; this was due to the presence of dense pelvic adhesions in these cases. One patient experienced an iatrogenic intestinal injury in the RALH group and three in the CL group. Vascular injury in the CL group was due to the umbilical trocar entry site, and vaginal bleeding occurred in the RALH group. However, hemostasis was achieved immediately. Intraoperative blood loss did not differ between groups, and no patient required a transfusion (Table 2). A conversion to laparotomy was not performed in either group.

According to the Clavien-Dindo classification, two first-grade complications occurred in the CL group and five first-grade complications in the RALH group. Of second-grade complications, five were recorded in the CL group, and three in the RALH group. Of third-grade complications, 11 occurred in the CL group and seven in the RALH group.

Postoperatively only one patient in the RALH group needed a revision in the operating room due to the formation of an abscess at the site of vaginal closure. Two patients in the CL group required a revision: one due to acute peritonitis as a result of iatrogenic intestinal injury, and the second as a result of abscess formation at the site of vaginal closure.

Fever was registered in six patients due to various reasons such as urinary tract infection, Clostridium difficile infection, or unknown causes. Paresthesia was recorded in two patients of the RALH group due to the long operating time and probably unsuitable positioning of the patients.

### 3.5. Length of Hospital Stay, Pain Scores, and Postoperative Intake of Painkillers

The mean duration of the patients’ hospital stay was 4.44 days (SD 3.214) in the RALH group and 4.13 days (SD 1.096) in the CL group. The mean postoperative pain score at one and four weeks after the operation were similar in the two groups: 3.26 (SD 2.809) and 1.19 (SD 1.733) in the CL group, and 2.73 (SD 2.136) and 1.11 (SD 1.385) in the RALH group, respectively.

Both groups consumed oral non-steroidal anti-inflammatory drugs for a similar period of time (median 4.00 days). No statistically significant difference was noted between groups with regard to any of these outcomes (Table 3).

### 3.6. Postoperative Satisfaction and Dissatisfaction

Ninety-six percent (*n* = 115) of the patients would recommend the operation to others under similar circumstances; RALH fared slightly better (100% *n* = 45) than CL (94.6% *n* = 70) in this regard. On a satisfaction score, 68% (*n* = 81) of all patients were highly satisfied with the treatment while 23.5% percent (*n* = 28) were satisfied. Six percent were moderately satisfied and one patient (0.8%) was dissatisfied. Seventy percent (*n* = 52) and 64% (*n* = 29) were highly satisfied in the CL and RALH groups, respectively. No significant differences between groups were noted with regard to any of these outcomes (Table 4).

### 3.7. Dissatisfaction with the Abdominal Incision

In whole 90.8% (*n* = 108) were total satisfied with the cosmetic result of the abdominal incision; the numbers in the respective groups were 80% (80% *n* = 36) in RALH and 97.3% (*n* = 72) in CL. The difference was significant on the Chi-square test (*p* = 0.002).

The detailed interview with the patients showed that 5.9% (*n* = 7) were dissatisfied with the scar; this was true of 13.3% (*n* = 6) in the RALH group and 1.4% (*n* = 1) in the CL group. The position of the incisions was a source of dissatisfaction for 1.7% (*n* = 2), which was true of 4% (*n* = 2) in the RALH group and no patient in the CL group (Table 5).

### 3.8. Limitation of Sexual Intercourse

Only 33% (*n* = 28) experienced no limitation of sexual intercourse after the operation. The two groups needed a similar period of time to resume sexual intercourse after the operation; the median time was 56 days for CL and 49 days for RALH.

**Figure 4:**
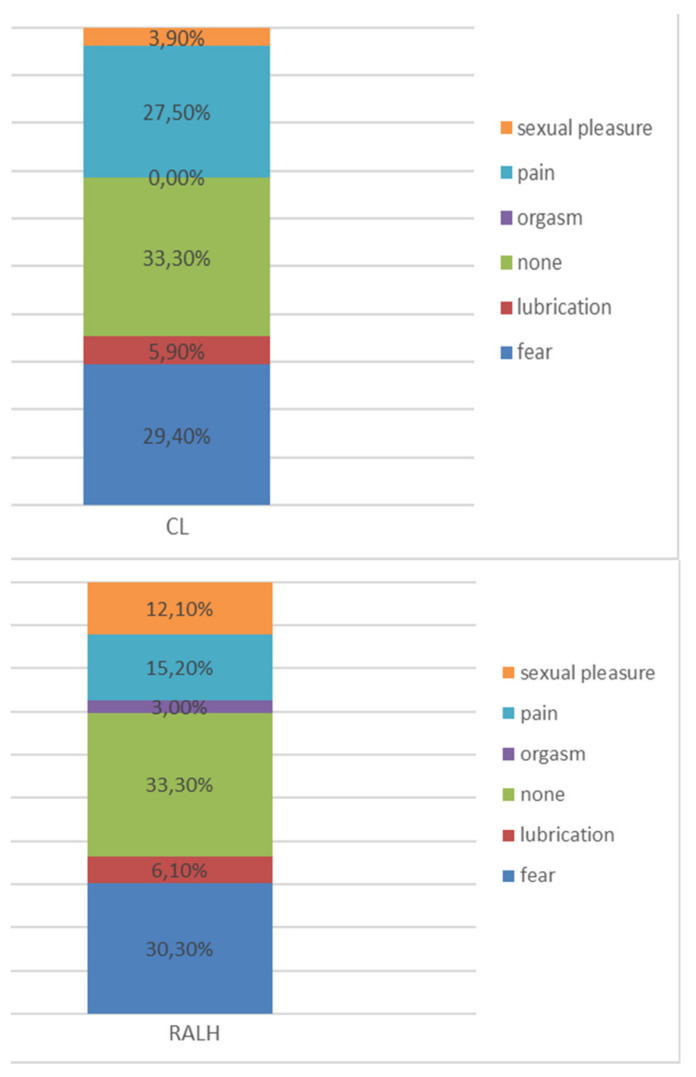
Limitation of sexual intercourse after the operation. Fear and pain were the most frequent limitations.

Nearly 30% (29.8% n = 25) were afraid to resume intercourse after the operation; no percentile difference was noted between groups. Pain was reported as a limitation by 22.6% (n = 19) in both groups. RALH was slightly superior to CL in regard of pain during sexual intercourse (15.2% n = 5, and 27.4% n = 14, respectively) (Figure 4).

### 3.9. Convalescence

The median number of days before starting to work after the operation was 42 days in both groups. The same numbers of days were needed by both groups to resume their hobbies. No statistical significance was noted between groups.

## 4. Discussion

The two groups investigated in the present study were similar in regard of age, body mass index, and indications for surgery. We also observed no significant differences in postoperative pain, the use of painkillers, intraoperative and postoperative complications.

We focused our analysis on clinical factors rather than economic aspects of the operation. The cohort studies published by Rosero and Wright report similar morbidity profiles for laparoscopic and robotic-assisted hysterectomy, with slightly higher costs for the latter procedure. However, other factors such as body mass index, uterine weight, and previous abdominal surgery were not addressed in earlier studies [19,20].

We maintained a three-week interval between sending the questionnaires and contacting patients on the phone for the interview. To minimize the possibility of patients trying to please the surgical team, the person conducting the telephone interview was not a member of the department.

The present study revealed that 20% of patients were dissatisfied with the abdominal incision in the RALH group, and a mere 2.7% in the CL group. This was most likely due to the rigidity of the RALH trocars compared to the disposable trocar used for CL. We conclude that greater attention should be given to counseling patients about the number of the scars and their positions. It might be feasible to develop a single incision port for robotic surgery and use a limited number of trocars.

Quality of life parameters such as pain scores at 1 and 4 weeks postoperatively, the period of taking painkillers, and the duration of convalescence were similar in the two groups. This concurs with a meta-analysis of prospective trials [21]. The question as to whether the patients would recommend the operation to others yielded a score of 100% for RALH and 94.6% for CL.

Difficulties in resuming sexual intercourse after the operation were experienced by 66.7% of our patients. Anxiety about resuming sexual intercourse was experienced by 30% in both groups. The time period from surgery to the resumption of sexual intercourse was four months. We conclude that patients should be counseled in detail about this aspect postoperatively. Recent studies published by Berlit show that the patients’ expectations concerning sexual function appear to influence postoperative outcomes. Therefore, this aspect as well as other personal factors should be considered when counseling patients [22,23].

Less invasive surgical methods of hysterectomy, such as those by the vaginal and laparoscopic approach tend to have a less destructive effect on sexual function [24]. Ercan suggested that, probably because of the positive effects of less invasive procedures on the patients’ self-esteem and quality of life, the procedures may be associated with no visible abdominal scar and a shorter recovery period [24]. Bastu and co-workers studied patients who underwent laparoscopic hysterectomy and those who underwent vaginal cuff closure; the authors found that although sexual function did not differ significantly preoperatively and three months postoperatively, vaginal lengths were significantly longer in the laparoscopic group [25]. In 2014 De La Cruz published a comparison of 38 total vaginal hysterectomies and 46 robotic hysterectomies, both of which were accompanied by pelvic support surgery, with regard to vaginal length and postoperative sexual functions. The authors registered no difference in sexual function, but a greater reduction in vaginal length after vaginal hysterectomy [26]. Therefore, when planning a laparoscopic hysterectomy, it would be advisable to opt for the laparoscopic cuff closure technique rather than the vaginal route in order to preserve vaginal length, to avoid alterations in the female sexual function [27].

In our study population, the median operating time was significantly longer in patients undergoing RALH compared to CL. This agrees with a Cochrane review published by Lawrie, which reported observational data on robotic-assisted and laparoscopic hysterectomy; operating times of about 1 to 2.5 h were noted for CL, and 3 h for RALH [4]. However, recently Lönnerfors registered data at university hospitals in Sweden and reported similar operating times for CL and RALH; the procedures were performed by a highly experienced robotic surgeon [28].

A variety of methods have been used in published studies to record operating time and operating theater time. One of disadvantages of our study is the absence of a mandatory log for all robotic cases, which calls for the documentation of port placement, docking and de-docking of the robot, and console time. We registered the time taken from the insertion of the uterine manipulator until final closure of the abdominal incision for both procedures. As this encompassed the entire operating time, we believe this parameter did not affect the outcome.

According to Lenihan, the learning curve for robotic-assisted surgery depends on setup time, console time, and the number of cases needed to stabilize a surgeon’s operating time; about 50 cases are deemed necessary for this purpose [29]. Regardless of individual variables, the total time needed for each procedure remains the point of maximum interest.

We were able to achieve a plateau in the learning after 30 cases. After 30 cases, the surgeon needed 133.74 min for the procedure. Our findings are consistent with a similar retrospective study comprising 45 patients with benign indication [30], and two further retrospective studies in which a significant improvement in operating time was noted after 20 robotic-assisted cases [31,32]. The fact that we were unable to achieve a plateau shortly before 30 cases was probably due to the presence of diverse surgical staff members at the beginning of the operation. The initial setup time in robotic surgery takes longer than the conventional laparoscopic approach, which can largely be overcome by adhering to a consistent and committed team of staff members in the operating room.

The shared decision for the route of the hysterectomy is influenced by various factors, including the indication of hysterectomy, adequate consultation of patients, and the surgeon’s level of training. Vaginal hysterectomy is primarily performed in conjunction with surgery to treat prolapse-disorders. The rate of vaginal hysterectomy in the United States decreased from 22% in 2003 to 19% in 2009–2010, which coincides with the introduction of robotic-assisted surgery [19,33].

Despite guidelines supporting the use of minimally invasive hysterectomy procedures, benign gynecological disease is still most commonly managed via laparotomy [19]. Our analysis suggests that, over a 4-year period, robotically assisted hysterectomy was used increasingly often for benign gynecologic disease.

Position statements from various associations of gynecological laparoscopy have not clearly endorsed the role of robotic assistance in laparoscopic hysterectomy. Further data will be needed to determine the most appropriate evidence-based applications of this technology for the treatment of benign disease.

## Figures and Tables

**Table 1:** Characteristics of the study population, indications and complications by the hysterectomy operation.

Characteristics	CL (*n* = 99)	RALH (*n* = 56)	*p*-Value
Age (years), Mean	49,00	49.09	
Median (range)	47.0 (42.0–54.0)	47.0 (43.0–52.0)	0.907
BMI (kg/m^2^), Mean	27.78	29.53	
Median (range)	26.66 (22.65–32.42)	27.71 (24.16–31.98)	0.265
Operative time (min.), Mean	162.73	131.31	
Lenghts of stay (nights), Mean	4.44	4.13	
Median (range)	4.0 (3.0–5.0)	4.0 (4.0–4.0)	0.514
Uterine weight (g), Mean	210.14	185.64	
Median (range)	150.0 (86.0–262.0)	141.0 (94.0–206.25)	0.804
Indications			
benign, *n* (%)	73 (73.3%)	48 (85.7%)	0.083
leiomyomas, *n* (%)	43 (43.4%)	28 (50.0%)	0.431
gynecologic (pre)cancer, *n* (%)	26 (26.3%)	9 (16.1%)	0.145
abnormal bleeding, *n* (%)	9 (9.1%)	6 (10.7%)	0.743
endometriosis, *n* (%)	17 (17.2%)	13 (23.2%)	0.36
other indications, *n* (%)	4 (4.0%)	1 (1.8%)	0.654

**Table 2:** Intraoperative and postoperative complications.

Complications Number (%)	CL	RALH	*p*-Value
	15 (15.2%)	12 (21.4%)	0.322
**Intraoperative**	8 (8.1%)	5 (8.9%)	0.999
Injuries of the urinary tract, *n* (%)	4 (4.0%)	2 (3.6%)	>0.999
Injuries of the gastrointestinal tract, *n* (%)	3 (3.0%)	1 (1.8%)	>0.999
Vascular injuries, *n* (%)	1 (1.0%)	3 (5.4%)	0.135
Other complications, *n* (%)	1 (1.0%)	0	>0.999
**Postoperative**	8 (8.1%)	9 (16.1%)	0.126
Revision surgery, *n* (%)	2 (2.0%)	1 (1.8%)	>0.999
Wound complications, *n* (%)	2 (2.0%)	2 (3.6%)	0.62
Thromboembolic complications, *n* (%)	0	1 (1.8%)	0.361
Mortality, *n* (%)	0	0	>0.999
Other complications, *n* (%)	5 (5.1%)	5 (8.9%)	0.497

**Table 3:** Postoperative pain scores and intake of painkillers.

Postoperative	CL	RALH	*p*-Value
Pain score at week 1 (mean)	3.26 (SD 2.809)	2.73 (SD 2.136)	
Median (range)	3.00 (0.75–5.00)	3.00 (1.00–4.00)	0.519
Pain score- week 4 (mean)	1.19 (SD 1.733)	1.11 (SD 1.385)	
Median (range)	0.00 (0.00–2.00)	1.00 (0.00–2.00)	0.693
Intake of painkillers (days) (mean)	11.92 (SD 43.043)	8.44 (SD 10.874)	
Median (range)	4.00 (1.00–7.00)	4.00 (2.50–7.00)	0.471

**Table 4:** Patient’s satisfaction-score with the outcome of the treatment based on a construct-specific satisfaction scale from 1 to 6; 1 indicated extreme satisfaction and 6 indicated dissatisfaction.

			Surgical Procedure		Total
			CL	RALH	
**Satisfaction with the outcome of treatment**	1	Number (Percentage)	52 (70.3%)	29 (64.4%)	81 (68.1%)
	2	Number (Percentage)	15 (20.3%)	13 (28.9%)	28 (23.5%)
	3	Number (Percentage)	5 (6.8%)	3 (6.7%)	8 (6.7%)
	4	Number (Percentage)	0 (0.0%)	0 (0.0%)	0 (0.0%)
	5	Number (Percentage)	1 (1.4%)	0 (0.0%)	1 (0.8%)
	6	Number (Percentage)	1 (1.4%)	0 (0.0%)	1 (0.8%)
Total		Number (Percentage)	74 (100.0%)	45 (100.0%)	119 (100.0%)
Statistical test		Chi-square test			0.823

**Table 5:** Satisfaction- and dissatisfaction- score with the abdominal incision.

			Surgical Procedure		Total	Statistical Test
			CL	RALH		Chi-Square Test
Total satisfaction		Number (Percentage)	72 (97.3%)	36 (80.0%)	108 (90.8%)	
Main cause of cosmetic dissatisfaction	Position of the incisions	Number (Percentage)	0 (0.0%)	2 (4.4%)	2 (1.7%)	0.002
	Number of scars	Number (Percentage)	0 (0.0%)	1 (2.2%)	1 (0.8%)	
	Scar	Number (Percentage)	1 (1.4%)	6 (13.3%)	7 (5.9%)	
	Painful/sensitive scars	Number (Percentage)	1 (1.4%)	0 (0.0%)	1 (0.8%)	
Total		Number (Percentage)	74 (100.0%)	45 (100.0%)	119 (100.0%)	

## Appendix A

I. Questionnaire

The table presents the questionnaire sent to the study population.

**German speaking community**	**English speaking community**
(1) Zum Zeitpunkt der OP waren Sie: verheiratet □ in einer festen Beziehung □ ledig □ verwitwet □?	(1) What was your relationship status at the time of the operation? Married- in a committed relationship- single- widowed
(2) Wie war Ihr Zustand zum Zeitpunkt der OP? Waren Sie: Noch nicht in den Wechseljahren □ in den Wechseljahren □ durch die Wechseljahre durch □?	(2) What was your hormone status at the time of the operation? Premenopausal Perimenopausal Post menopausal
(3) Wie lange waren Sie krankgeschrieben nach der Gebärmutterentfernung? Tage__________________	(3) How long were you on sick leave after hysterectomy? Days: _____________________
(4) Auf einer Skala von 1-6, wobei analog zum Schulnotensystem, hier 1 = sehr zufrieden/bestmögliche Zufriedenheit und 6 = sehr unzufrieden/überhaupt keine Zufriedenheit, bedeutet: Wie zufrieden sind Sie dann mit dem Behandlungsergebnis der OP insgesamt? Skala: 🙂 1 □ 2 □ 3 □ 4 □ 5 □ 6 □ 🙁	(4) How satisfied are you with the result of the hospital treatment all in all? On a scale from 1–6, whereby analogous to the German school grading system, here 1 means = very satisfied/best possible satisfaction and 6 means = very dissatisfied/no satisfaction at all: Scale 🙂 1 □ 2 □ 3 □ 4 □ 5 □ 6 □ 🙁
(5) Würden Sie diese Operation unter den gleichen Umständen weiterempfehlen? Ja □ Nein □	(5) In the same circumstances, would you recommend this surgery to others? Yes □ No □
(6) Auf einer Skala von 0–10, wobei hier 0 = keine Schmerzen, 10 = stärkste vorstellbare Schmerzen bedeutet, Wie waren die Schmerzen nach der Operation im Abstand nach: 6a) Ca. 1 Woche? Skala: 🙂 0 □ 1 □ 2 □ 3 □ 4 □ 5□ 6 □ 7 □ 8 □ 9 □ 10 □ 🙁 6b) Ca. 4 Wochen? Skala: 🙂 0 □ 1 □ 2 □ 3 □ 4 □ 5 □ 6 □ 7 □ 8 □ 9 □ 10 □ 🙁	(6) How was the pain after surgery? On a scale of 0–10, 0 means = no pain, 10 means = strongest imaginable pain 6a) After 1 week: Scale: 🙂 0 □ 1 □ 2 □ 3 □ 4 □ 5 □ 6 □ 7 □ 8 □ 9 □ 10 □ 🙁 6b) After 4 weeks: Scale 🙂 0 □ 1 □ 2 □ 3 □ 4 □ 5 □ 6 □ 7 □ 8 □ 9 □ 10 □ 🙁
(7) Wie lange haben Sie Schmerzmittel nach der OP eingenommen? In Wochen:__________________	(7) How many weeks did you take pain medication after the operation?
(8) Wie schnell nach der OP sind Sie zum Alltag zurückgekehrt? Das heißt.: (8a) Wie viele Tage brauchten Sie Hilfe beim Anziehen, Waschen oder Benutzen der Toilette? (8b) Wie viele Tage waren Sie bei den tagtäglichen Beschäftigungen im Haushalt eingeschränkt? (8c) Wie viele Tage waren Sie bei Ihren Hobbys oder anderen Freizeitbeschäftigungen, inklusive Sport, eingeschränkt?	(8) How quickly did you return to everyday life after the operation? Specifically, we wanted to know: (8a) How many days did you need help getting dressed, washing or using the toilet? (8b) How many days were you restricted from daily activities in the household? (8c) How many days were you restricted in your hobbies or other leisure activities, including sports?
(9) Wenn Sie in einer festen Partnerschaft zum Zeitpunkt der OP waren: (9a) Nach wie vielen Tagen/Wochen hatten Sie wieder Geschlechtsverkehr? (9b) Wie viele Tage/Wochen waren Sie beim Geschlechtsverkehr, nachdem Sie wieder damit begonnen hatten, eingeschränkt? (9c) Was war am ehesten der Grund für die Einschränkung beim Geschlechtsverkehr? □ Schmerzen □ sexuelle Lust □ Lubrikation (Feuchte) □ Orgasmus □ Angst	(9) If you were in a permanent partnership at the time of the operation: (9a) After how many days/weeks did you have sexual intercourse again? (9b) How many days/weeks were you restricted in sexual intercourse after starting again? (9c) What was most likely the reason for the restriction in sexual intercourse? □ pain □ sexual pleasure □ lubrication (moisture) □ orgasm □ fear
(10) Sind Sie mit dem kosmetischen Ergebnis der Operation zufrieden? Ja □ Nein □ (10a) Wenn nein: Was ist am ehesten die Ursache der Unzufriedenheit? Ort der Narben □ Anzahl der Narben □ die Länge der Narben □ Beschaffenheit der Narben □ Schmerzhafte/empfindliche Narben □	(10) Are you satisfied with the cosmetic result of the operation? Yes □ No □ (10a) If not: What is most likely cause of the dissatisfaction? Location of scars □ number of scars □ length of scars □ Nature of the scars □ Painful/sensitive scars □
